# Effects of *Helicoverpa armigera* Egg Age on Development, Reproduction, and Life Table Parameters of *Trichogramma euproctidis*

**DOI:** 10.3390/insects12070569

**Published:** 2021-06-22

**Authors:** Nazanin Atashi, Parviz Shishehbor, Ali Asghar Seraj, Arash Rasekh, Seyed Ali Hemmati, Eric W. Riddick

**Affiliations:** 1 Department of Plant Protection, Faculty of Agriculture, Shahid Chamran University of Ahvaz, Ahvaz 61357-43311, Iran; Nazanin-Atashi@stu.scu.ac.ir (N.A.); pshishehbor@scu.ac.ir (P.S.); seraj.a@scu.ac.ir (A.A.S.); a.rasekh@scu.ac.ir (A.R.); sa.hemmati@scu.ac.ir (S.A.H.); 2 Agricultural Research Service, United States Department of Agriculture, Stoneville, MS 38776, USA

**Keywords:** biological control, endoparasitoid, life tables, natural enemy, reproduction

## Abstract

**Simple Summary:**

This study evaluated the potential of *Trichogramma* *euproctidis* to parasitize *Helicoverpa* *armigera* eggs of different ages in laboratory arenas. *H*. *armigera* is a major pest of agricultural crops in Iran and other countries. *T*. *euproctidis* is an important egg parasitoid of lepidopteran eggs and could be used in augmentative biological control of *H*. *armigera*. The objective of this research was to determine if young rather than old *H. armigera* eggs were optimal for *T*. *euproctidis* development, reproduction, and life table parameters. Results indicated that *T. euproctidis* developed faster and produced more offspring in 14 h old rather than 38 h or 62 h old *H*. *armigera* eggs. A life table analysis confirmed these results. This study is important because it documents for the first time that *T. euproctidis* can utilize *H*. *armigera* as a rearing host. Using young rather than old host eggs could ensure the persistence of a *T*. *euproctidis* mass production system to support augmentative releases.

**Abstract:**

The noctuid *Helicoverpa armigera* is an economically important pest of agricultural crops in Iran and other countries. Research is evaluating the capacity of *Trichogramma* parasitoids to control *H*. *armigera* populations on field crops. The objective of this research was to determine if young rather than old *H. armigera* eggs were optimal for *Trichogramma* *euproctidis* development, reproduction, and life table parameters. Bioassays involved exposing *T*. *euproctidis* mated females to *H*. *armigera* 14, 38, or 62 h old eggs within 24 h in laboratory arenas. Results indicated that the number of host eggs parasitized successfully by *T. euproctidis* decreased as host egg age increased. Host egg age had no significant effect on *T*. *euproctidis* adult emergence. Adults that developed in 14 h old eggs had greater longevity and fecundity than those that developed in 38 h or 62 h old eggs. The intrinsic rate of increase (*r*) was greatest, and the mean generation time (T) was lowest for *T. euproctidis* reared in 14 h old eggs. This study indicates that young *H*. *armigera* eggs are more suitable than old ones for *T*. *euproctidis* development and reproduction. This study is important because it provides evidence, for the first time, that *T. euproctidis* can utilize *H*. *armigera* as a rearing host. Using young rather than old host eggs could ensure the persistence of a *T*. *euproctidis* mass production system to support augmentative releases.

## 1. Introduction

*Helicoverpa armigera* (Hübner) (Lepidoptera: Noctuidae) is an important pest of agricultural crops around the world. If uncontrolled, it can significantly reduce production and yield of cotton (*Gossypium hirsutum* L.) in northeast Iran and tomato (*Lycopersicon esculentum* Mill.) in southwest Iran [1,2,3,4,5]. Synthetic chemical insecticides play an important role in the management of *H*. *armigera*. However, resistance of *H. armigera* to conventional insecticides has been reported in some countries such as Australia [5], China [6], India [7], Pakistan [8], Turkey [9], Iran [10], and France [11]. Using biological control as an alternative to conventional insecticides to manage *H*. *armigera* should be encouraged [12]. The development of techniques to conserve and utilize indigenous natural enemies, particularly predators and parasitoids that attack egg and larval stages, could be critical to successful biological control of *H*. *armigera* [12]. In addition, mass rearing and releasing the most important natural enemies could boost the chances of controlling *H*. *armigera*.

*Trichogramma* species (Hymenoptera: Trichogrammatidae) are commonly deployed to attack the egg stage of lepidopteran pests all over the world [13,14,15,16]. *Trichogramma euproctidis* (Girault), previously known as *T*. *turkestanica* Meyer [17], parasitized a noctuid *Trichoplusia ni* (Hübner), a plutellid *Plutella xylostella* (L.), and a pyralid *Ephestia kuehniella* Zeller in a laboratory study [18]. Large host eggs usually yielded larger-sized and more fecund *T*. *euproctidis* females [18]. Moreover, the size of hind tibia of *T*. *euproctidis* adult females was correlated to egg size (volume) of the hosts from which they emerged. Large *T*. *euproctidis* females emerged from *T*. *ni* eggs, which were the largest of the three host species; small adults emerged from *P*. *xylostella* eggs, which were the smallest of the host species. *T*. *euproctidis* progeny size (egg volume) was greatest for females that emerged from *T*. *ni* eggs [18]. In field studies, *T*. *euproctidis* parasitized the gelechiid *Tuta absoluta* (Meyrick), a major pest of tomato in greenhouses in Egypt [19,20].

Many factors such as host egg age affect the success of an egg parasitoid [21,22]. Young host eggs are usually preferred over old eggs. For example, *Trichogramma cacoeciae* Marchal parasitized more 1–2 d old than 3–4 d old hosts, *Lobesia botrana* Denis and Schiffermüller (Lepidoptera: Tortricidae), in the laboratory [15]. Note that young rather than old (black head) eggs of *Diatraea grandiosella* Dyar (Lepidoptera: Crambidae) were preferred as a host by *Trichogramma pretiosum* Riley [23]. In the cases where old *D*. *grandiosella* eggs were parasitized, egg yolk was still present and *T*. *pretiosum* eggs were always located within it [23]. *Trichogramma* larvae are semi-liquid feeders that use extra-oral and intra-oral digestion to consume host egg yolk and other egg materials [24,25]. Host eggs become less suitable as they get older, possibly because they contain less yolk or because they have a thicker cuticle (chorion), which could limit envenomation of host eggs by *Trichogramma* females [26]. These laboratory studies suggest that *Trichogramma* mass rearing efforts would be more successful when young, rather than old, host eggs were provided as hosts. 

Although several *Trichogramma* species are known to parasitize *H*. *armigera* eggs in the field [27,28] or laboratory [29,30,31], *T. euproctidis* is not one of them. Therefore, the aim of this study is to provide baseline information on parasitism of *H*. *armigera* and its suitability as a host for *T*. *euproctidis* in the laboratory. The specific objective of this research is to determine if young rather than old *H. armigera* eggs are optimal for *T*. *euproctidis* development, reproduction, and life table parameters. This research should provide clues to developing efficient mass rearing systems for *T*. *euproctidis*. 

## 2. Materials and Methods

### 2.1. Insect Collection

A colony of *T. euproctidis* was originally obtained by pasting *E. kuehniella* egg masses on white paper (5 cm length, 1 cm width) then attaching to foliage in a corn field on the premises of Shahid Chamran University of Ahvaz (31°17′59″ N, 48°39′39″ E), Ahvaz, Iran, during July 2019. The species was identified using SEM micrographs of male genital capsules [32]. Voucher specimens were deposited in the collection of Department of Plant Protection, Shahid Chamran University of Ahvaz, Ahvaz, Iran, and the Natural History Museum, London, UK. The colony of *T. euproctidis* was maintained using the eggs of *E. kuehniella* under conditions of 25 ± 1 °C, 55 ± 5% RH, with a 16:8 L/D regime. For mass rearing of *Trichogramma* species, *E*. *kuehniella* eggs are often used as hosts [13] because they only support the complete development of one parasitoid, rarely two, after emergence [13]. The *E. kuehniella* eggs used in this study were obtained from a colony maintained at Golestan Mooud Insectary Company, Ahvaz, Iran. *T*. *euproctidis* was reared from *E*. *kuehniella* eggs for five to six generations in the laboratory prior to experimentation. 

*Helicoverpa armigera* eggs were obtained from a laboratory culture reared on a bean-yeast based semi-artificial diet [33] at the University of Guilan, Rasht, Iran, and reared continuously on the same diet at 25 ± 1 °C, 55 ± 5% RH, and a photoperiod of 16:8 (L/D). After emergence, *H*. *armigera* females were confined with potted geranium, *Pelargonium zonale* (L.), 2-month-old seedlings in a cage (diameter 50 cm, height 40 cm) for oviposition. Eggs laid on geranium leaves within a 40-min period were maintained at 25 ± 1 °C until they were either 14, 38, or 62 h old. The midpoint of the 40-min period was used as the starting point for determining egg age.

### 2.2. Experimental Procedure

A preliminary experiment showed that at 25 °C, *H. armigera* eggs take less than 4 d to hatch (N.A. and P.S., *unpublished data*). Consequently, 14 h, 38 h, and 62 h old *H. armigera* eggs were selected as test hosts. The newly emerged *T. euproctidis* adult females were confined with males for 8 h, then introduced into a clear glass tube (diameter 1 cm, height 10 cm) containing a *H. armigera* egg mass. The glass tubes were sealed with cotton wool. Each egg mass of *H. armigera* used in our experiments contained 40 eggs. Egg masses were glued on a piece of white paper (5 cm length, 1 cm width). Parasitoids had not been in contact with host eggs before the tests. Female parasitoids were fed with droplets of honey deposited in the inner wall of each tube during the experiments. The female parasitoid was allowed to parasitize *H. armigera* eggs in a growth chamber (25 ± 1 °C, 55 ± 5% RH, 16:8 L/D) for 24 h. The parasitoid was removed after 24 h and tubes were kept in the incubator until all parasitoid progeny emerged. Ten replications were used for each egg age. The experiment was carried out in a completely randomized design. The number of parasitized *H. armigera* eggs from each glass tube was determined under a stereo zoom microscope six days after the parasitoid was removed. The date of emergence of *T. euproctidis* adults was recorded to estimate the duration of pre-imaginal development. In addition, survival rate (percentage of adult emergence), number of parasitoids emerged per egg, the sex of emerging wasps, and the size of right hind tibia (as an estimate of body size [34,35]) of female and male parasitoids were recorded using a stereo zoom microscope.

The effect of host egg age on longevity and fecundity of adult progeny was studied by placing one adult female and one adult male (less than 24 h old) in a glass tube (as in tubes described in the previous experiment) containing an egg mass of 40, 1 d old *H. armigera*, and honey. Males were replaced in case they died early in the experiment. New eggs were offered daily to each female until all females were dead. The experiment was carried out in a completely randomized design. This experiment was also conducted in a growth chamber (25 ± 1 °C, 55 ± 5% R.H. and 16:8 L/D). Longevity and fecundity of parasitoids were calculated. Those females which were injured during daily handling or those that died because of getting stuck in honey droplets were excluded from data analysis.

### 2.3. Statistical Analysis

All data on number of host eggs parasitized, percentage survival rate, developmental time of offspring, percentage of female progeny, number of parasitoids per host egg, size of hind tibia, and longevity and fecundity of progeny were tested for normality using the Kolmogorov–Smirnov test. The data were analyzed using one-way analysis of variance (ANOVA). Percentage survival rate and percentage of female progeny were arcsine transformed to homogenize variances prior to the one-way ANOVA. Mean values were separated using Tukey’s honest significant difference (HSD) test at α = 0.05. SPSS version 22 statistical software was used to analyze the data [36].

Life table parameters were estimated by combining data from the pre-imaginal development, adult survival, and reproduction experiment of different host egg age treatments. The intrinsic rates of population increase were estimated by iteratively solving the following equation: *Ʃ*e *^r^*^x *l*x *m*x^ = 1 [37], where x is the mean age class, *m*_x_ is the mean number of female progeny per female of age x, and *l*_x_ is the probability of survival to age x. A trial number of values for *r* were substituted into the equation until the *r* value for which the sum on the left side of the equation approximates unity. The Jacknife procedure was used to estimate an SE for the *r* values of different treatments [38]. Further data were also calculated for each treatment: net reproductive rate (*R*_0_ = *Ʃ l*_x_ *m*_x_, number of female offspring produced per female), mean generation time (T = Ln *R*_0_/*r*), doubling time (DT = ln 2/*r*, number of days required for the population to double in numbers), and finite rate of increase (λ = e*^r^*, number of times the population will multiply itself per unit of time) [37]. Standard error of the population growth parameters was achieved by using the bootstrap technique, and multiple comparisons were made possible using the paired bootstrap test with 100,000 samples.

## 3. Results

Host age had a significant effect on the mean number of eggs parasitized by *T*. *euproctidis* (F = 32.43; df = 2, 27; *p* < 0.0001) (Table 1). The number of parasitized eggs decreased as egg age increased. Mean number of 14 h old eggs parasitized by *T*. *euproctidis* was two times higher than for 38 h and 62 h old eggs. Preimaginal developmental times for *T*. *euproctidis* were significantly affected by host age (females: F = 190.82; df = 2, 393; *p* < 0.0001; males: F = 35.38; df = 2, 233; *p* < 0.0001) (Table 1). Developmental time of *T*. *euproctidis* females and males were shortest on 14 h old host eggs. There was no significant difference in development time of males and females between 38 h and 62 h old host eggs. The host egg age had no significant effect on survival rate (adult emergence) (F = 2.46; df = 2, 27; *p* = 0.104) (Table 1).

Sex ratio (proportion of female progeny) was significantly affected by host egg age (F = 4.81; df = 2, 27; *p* = 0.016) (Table 1). A female biased sex ratio was observed on all egg ages tested, but percentage of female progeny declined as *H*. *armigera* egg age increased. The host egg age had a significant effect on size of right tibial length of emerging parasitoids (females: F = 204.61; df = 2, 212; *p* < 0.0001; males: F = 57.82; df = 2, 141; *p* < 0.0001; Table 1). Size of right tibial length of parasitoids steadily decreased as host egg age increased. The number of parasitoids emerging from each host egg was significantly affected by the age of the host (F = 3.51; df = 2, 27; *p* = 0.04) (Table 1).

Host age had a significant effect on female longevity (F = 148.87; df = 2, 87; *p* < 0.001) (Table 2). The longest-lived *T*. *euproctidis* females emerged from 14 h old eggs (6.92 d). The mean daily and total number of eggs oviposited by *T*. *euproctidis* females emerging from different host ages were significantly different (daily fecundity: F = 53.79; df = 2, 87; *p* < 0.001; total fecundity: F = 67.53; df = 2, 87; *p* < 0.001) (Table 2). Female *T*. *euproctidis* oviposited a mean number of 67.73 eggs on 14 h old *H*. *armigera* eggs compared to 19.50 eggs on 62 h old host eggs.

**Figure 1:**
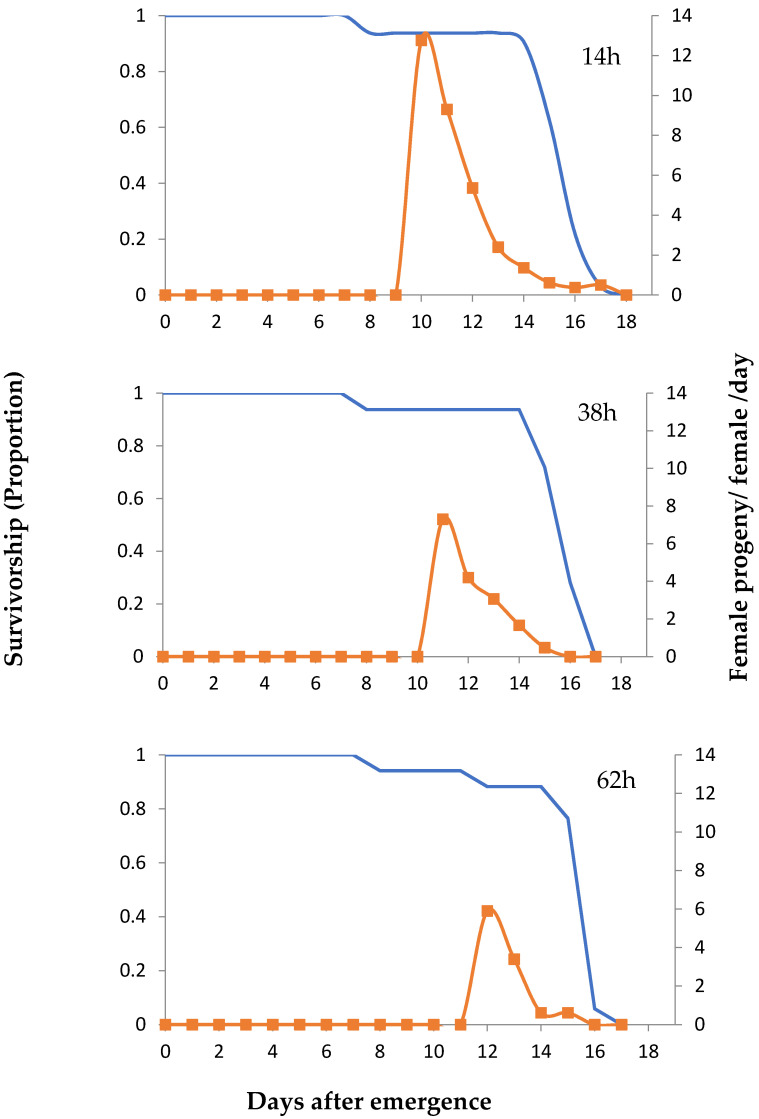
Proportional survivorship (*l*_x_, solid blue lines) and number of *T*. *euproctidis* female progeny per female per day (*m*_x_, dotted orange lines) from *H. armigera* eggs of different ages.

Life table parameter statistics emphasized that 14 h old eggs were a preferable host egg age for *T*. *euproctidis* compared with other host ages tested (Table 3). As a result of accelerated development duration and greater fecundity early in adulthood, *T*. *euproctidis* reared on 14 h old eggs had a significantly greater gross reproduction rate (GRR), net reproduction rate (R_0_), and intrinsic rate of natural increase (*r*) than those bred on other host egg ages tested (Table 3). In addition, the shortest and longest mean generation times of *T*. *euproctidis* were obtained on the 14 h old eggs and 62 h old eggs, respectively. Age-specific survival (*l*_x_) and fecundity (*m*_x_) curves derived from these data for each tested host egg age are illustrated in Figure 1.

## 4. Discussion

In this study, all *H. armigera* eggs were accepted by *T. euproctidis* females as suitable hosts. However, 14 h old eggs were parasitized at a twofold higher rate than the other egg ages, suggesting a preference for younger eggs. *T. euproctidis* was able to develop faster in young rather than old host eggs. *Trichogramma* species often develop and survive better on young host eggs [39], perhaps because essential nutrients in the host egg are progressively incorporated into and assimilated by the host embryo, becoming less available to support parasitoid development [40]. Moreover, *Trichogramma* species are occasionally unable to complete development inside old host eggs [41], possibly due to the rotation of the host embryo or sclerotization of its head capsule [23]. Lepidopteran eggs that have completed 75% or more of their embryonic development are not suitable for *Trichogramma* development [23].

The age of the *H*. *armigera* host egg did not affect survival rate (emergence rate) of *T*. *euproctidis* offspring. Similar results were also reported for *Trichogramma chilonis* Ishii emerging from *Plutella xylostella* (L.) [42], *Trichogramma cacoeciae* Marchal, *Trichogramma principium* Sugonjaev & Sorokina, and *Trichogramma evanescens* Westwood from *Phythorimaea operculella* (Zeller) [43], *T. cacoeciae* from *Lobesia botrana* Denis and Schiffermuller [15], *T. pretiosum* from *Mocis latipes* (Guenee) [44], and *Trichogramma galloi* Zucchi from *Diatraea saccharalis* (F.) [45]. However, *T. evanescens* offspring survival rate declined as *E*. *kuehniella* egg age increased. On the basis of these studies, we found that lack of host age effects on parasitoid survival is the norm rather than the exception in *Trichogramma* species.

Variable results have been reported on the impact of host egg age on sex ratio of progeny of *Trichogramma* species [23]. The age of *H. armigera* eggs had a significant effect on *T. euproctidis* progeny sex ratio, and it should be noted that the sex ratio was biased toward females for all host egg ages tested. Sex ratio (percentage of female progeny) declined when *T. euproctidis* parasitized older *E. kuehniella* eggs [22]. Moreover, *E. kuehniella* and *Sitotroga cerealella* Olivier egg ages had a significant effect on sex ratio of *T. evanescens*; younger eggs produced more female parasitoids [46]. However, host egg age did not affect the sex ratio of *T. pretiosum* (Riley) reared from *D. grandiosella* [31], *T. principium* from *S. cerealella* [47], *T. brassicae* from *Trichoplusia ni* (Hübner) and *Pieris rapae* (L.) [39], *Trichogramma dendrolimi* Matsumura from *Mamestra brassicae* (L.) [48], *T. cacoeciae* from *L. botrana* [49], *Trichogramma fuentesi* Torre from *Cactoblastis cactorum* (Berg) [50], and *T. dendrolimi* from *Chilo suppressalis* (Walker) [51]. These differences may be explained by differences in *Trichogramma* species, host species, or experimental conditions.

Parasitoid body size was affected by the age of host eggs. Female *T*. *euproctidis* right tibia were longer upon emergence from young rather than old *H*. *armigera* eggs. Similarly, *T. pretiosum* tibial length was greatest on individuals emerging from young rather than old *H. zea* eggs [40]. Tibial length is commonly used as a measure of body size; longer tibia positively correlates with larger body size of parasitoids [34,35]. Body size has a direct relationship with female longevity and/or fecundity of egg parasitoids; larger females typically live longer and produce more progeny [52,53]. Therefore, the age of the host eggs in which *T. euproctidis* develop may have an immediate effect on developmental time and a long-term effect on female fitness. 

Host egg age had a significant effect on the number of parasitoids that developed per egg, and the number of offspring that emerged per egg was continuously more than one progeny per parasitized egg. This was attributed to the size of *H. armigera* eggs, which were from 0.42 to 0.60 mm [54] compared to the eggs of the factitious host *E. kuehniella,* which were approximately 0.3 mm [55]. These findings suggest that availability of a larger amount of nutrients (i.e., yolk) in the host egg could support the development of more than one parasitoid. However, when more than one parasitoid develops in the same host egg, there may be competition for nutrients resulting in smaller offspring, which in turn result in lower longevity and fecundity of emerged parasitoids [56].

At 25 °C, mean longevities of *T. euproctidis* females parasitizing 1 d old *E. kuehniella* eggs were 8.9 d [57] and 9.8 d [22] compared to 6.9 d for females parasitizing 1 d old *H*. *armigera* eggs in this study. Mean total fecundities of *T. euproctidis* parasitizing 1 d old *E. kuehniella* eggs were 82.1 eggs [57] and 78.9 eggs [22] at 25 °C in comparison to 67.7 eggs for females parasitizing *H*. *armigera* eggs in this study. Differences in host species and experimental conditions (e.g., humidity) could account for the lower fecundity of *T. euproctidis*. The rearing history of *T*. *euproctidis* could have affected fecundity. The *T*. *euproctidis* colony was maintained on *E*. *kuehniella* eggs for several generations prior to beginning this experiment. Laboratory adaptation to the rearing host could partly explain why *T*. *euproctidis* females were more fecund when reared from *E*. *kuehniella* eggs (in previous studies) rather than *H. armigera* eggs, in this study. Future research could include both host species, i.e., factitious versus new host, in the same experimental design to test the influence of laboratory adaptation on *T*. *euproctidis* fecundity. 

Host egg age affected the biological performance of *T. euproctidis*, as demonstrated in its intrinsic rate of increase (*r*). Intrinsic rate of increase reflects survival and reproduction of the parasitoid [58]. The highest *r* value (0.311/d) was recorded for progeny produced in 14 h old host eggs. At the same temperature, *T. euproctidis* had a *r* value of 0.354/d from the same egg age, i.e., 24 h old *E. kuehniella* eggs [22]. The discrepancy between the results of the mentioned study and this study may be due to differences in the size of host species. 

In conclusion, *T. euproctidis* developed faster in 14 h old *H*. *armigera* eggs and produced significantly more offspring than other egg ages tested. Although *T*. *euproctidis* developed and emerged successfully from all *H*. *armigera* egg ages, the outcome was decreased female progeny, decreased body size of progeny, and decreased population growth rate when older *H*. *armigera* eggs were utilized as hosts. The long-term fitness of a *T*. *euproctidis* colony would decline. Therefore, this study suggests that young-aged *H*. *armigera* eggs be used in a system designed to mass produce *T*. *euproctidis* for augmentative releases. Moreover, this study could suggest that *T*. *euproctidis* adults should be released as soon as *H*. *armigera* adults are first detected in crop fields. This would increase the likelihood that *T*. *euproctidis* females locate and parasitize young rather than old *H*. *armigera* eggs. This strategy could improve the persistence of *T*. *euproctidis* in the field during the growing season.

## Figures and Tables

**Table 1:** Parasitism and biological parameters of *T*. *euproctidis* emerging from *H. armigera* eggs of different ages. Data represent mean (±SE) values.

Parameter	*H*. *armigera* Egg Age (h)		
	14	38	62
Number of parasitized eggs	30.80 ± 2.18 ^a^	15.0 ± 1.77 ^b^	11.60 ± 1.34 ^b^
Female development time (d)	10.07 ± 0.05 ^c^	11.57 ± 0.08 ^b^	12.07 ± 0.10 ^b^
Male development time (d)	10.28 ± 0.07 ^b^	11.26 ± 0.12 ^a^	11.41 ± 0.19 ^a^
Survival rate (%)	94.10 ± 1.38 ^a^	93.4 ± 1.79 ^a^	88.50 ± 2.49 ^a^
Sex ratio (% female)	73.70 ± 3.99 ^a^	64.70 ± 3.45 ^b^	59.80 ± 3.72 ^c^
Size (mm) of right hind tibia (female)	0.180 ± 0.056 ^a^	0.172 ± 0.085 ^b^	0.160 ± 0.080 ^c^
Size (mm) of right hind tibia (male)	0.170 ± 0.073 ^a^	0.160 ± 0.092 ^b^	0.150 ± 0.030 ^c^
Number of parasitoids/host egg	1.2 ± 0.45 ^a^	1.1 ± 0.36 ^ab^	1.03 ± 0.43 ^b^

^abc^ Mean values in a row followed by the same letters are not significantly different at *p* > 0.05 (Tukey’s HSD test).

**Table 2:** Mean (± SE) oviposition period, female and male longevity (d), and diel and total fecundity of *T. euproctidis* reared from *H.* *armigera* eggs of different ages.

Parameters	*H*. *armigera* Egg Age (h)		
	14	38	62
Oviposition period (d)	5.19 ± 0.237 ^a^	4.46 ± 0.124 ^b^	3.62 ± 0.090 ^c^
Female longevity (d)	6.92 ± 0.159 ^a^	5.017 ± 0.122 ^b^	3.99 ± 0.70 ^c^
Male longevity (d)	4.59 ± 0.183 ^a^	2.86 ± 0.124 ^b^	1.829 ± 0.128 ^c^
Diel fecundity	10.83 ± 0.657 ^a^	6.84 ± 0.67 ^b^	5.32 ± 0.36 ^b^
Total fecundity	67.73 ± 4.33 ^a^	31.90 ± 3.73 ^b^	19.5 ± 2.91 ^c^

^abc^ Mean values in a row followed by the same letters are not significantly different at *p* > 0.05 (Tukey’s HSD test).

**Table 3:** Mean (±SE) values of life table parameters for *T*. *euproctidis* reared from *H*. *armigera* eggs of different ages.

Parameters	*H*. *armigera* Egg Age (h)		
	14	38	62
GRR (offspring/Generation)	32.6904 ± 0.1041 ^a^	16.7116 ± 0.0482 ^b^	10.5308 ± 0.274 ^c^
R_0_ (offspring/Generation)	30.1514 ± 0.091 ^a^	14.8696 ± 0.041 ^b^	9.2195 ± 0.023 ^c^
*r* (d^−1^)	0.3112 ± 0.00026 ^a^	0.2288 ± 0.00023 ^b^	0.1771 ± 0.0002 ^c^
λ (d^−1^)	1.3651 ± 0.0003 ^a^	1.2571 ± 0.0029 ^b^	1.1938 ± 0.0002 ^c^
T (d)	10.9437 ± 0.0016 ^c^	11.7974 ± 0.0028 ^b^	12.5409 ± 0.0012 ^a^
DT (d)	2.2271 ± 0.0018 ^c^	3.0296 ± 0.0031 ^b^	3.9136 ± 0.0044 ^a^

^abc^ Values in rows followed by the same small letter are not significantly different using the paired bootstrap test at 5% significance level. Note that *r* is the intrinsic rate of increase, λ is finite rate of increase, T is mean generation time in days, and DT is doubling time in days.

## Data Availability

The authors can provide supporting data on ResearchGate.
